# History of Dentistry

**Published:** 1874-05

**Authors:** J. Taft


### HISTORY OF DENTISTRY.

A "History of Dentistry" as it has been developed in the United States, is being prepared, and will be published so soon as the material for the work can be obtained and put into proper shape.

The facility with which this can be done will depend much upon the aid and co-operation which the profession will give. Quite a number have furnished material already, and many others have promised to do so, and are preparing to fulfill the promise.

There is a large amount of material, historical in character, in possession of individuals in the profession, that ought to be included in such a work, both as matter of interest, and justice too. There are many things passing by, and that will soon be forever gone, especially so far as their origin is concerned, unless they are put upon proper record; and it is for the accomplishment of this purpose, that the "History of Dentistry" is being prepared, and NOT for indulging "in a species of self-adulation and personal advertisement." The subjects to be embraced in this work are, an account of dentistry from its beginning in this country to the present time, ncluding a history of dental associations, as exhibited in the

American Society of Dental Surgeons, the American Dental Convention, American Association, together with some account of State Societies. And an account of the professional journalism, giving a history of each periodical. Also a history of the textual literature of the profession. A history of the colleges of the profession, in which should be embraced an account of their organization and work accomplished. In all these things, giving such particulars as truth and justice require. In addition to these subjects, the different modes of practice that have been from time to time employed will be presented; as also instruments, appliances and materials used.

Now in reference to all these things, we shall be pleased to receive communications; descriptive and historical, full, concise and comprehensive.

Some criticism has been indulged, in reference to the biographical department. The difficulties that attach to this work are as fully apprehended by those engaged in it as any body else, and it is of quite as much importance to them as to any others. The present indications are that when a complete history is prepared, as above suggested, there will be very little material left of which to make biographies. If anything at all is done in that direction, it will be chiefly of those who passed away, and if of any living men, of those whose professional life has about closed, and of whom something might properly be said, after a history of dentistry has been written.

There will be no "self-adulation and advertisement of men whose professional attainments are inferior;" so all anxiety may be dismissed on that score. This work will be valuable in a large measure as it shall receive the co-operation of the best men of the profession.

We shall be glad to receive interesting and valuable contributions, bearing upon any of the subjects for this work.

J. Taft.

